# Identification of *Dermatophyte* Species after Implementation of the In-House MALDI-TOF MS Database

**DOI:** 10.3390/ijms150916012

**Published:** 2014-09-11

**Authors:** Adriana Calderaro, Federica Motta, Sara Montecchini, Chiara Gorrini, Giovanna Piccolo, Maddalena Piergianni, Mirko Buttrini, Maria Cristina Medici, Maria Cristina Arcangeletti, Carlo Chezzi, Flora De Conto

**Affiliations:** Unit of Microbiology and Virology, Department of Clinical and Experimental Medicine, University of Parma, Viale A. Gramsci 14, Parma 43126, Italy; E-Mails: fmotta@ao.pr.it (F.M.); sara.montecchini@unipr.it (S.M.); cgorrini@ao.pr.it (C.G.); giovanna.piccolo@unipr.it (G.P.); maddalena.piergianni@nemo.unipr.it (M.P.); mirko.buttrini@unipr.it (M.B.); mariacristina.medici@unipr.it (M.C.M.); mariacristina.arcangeletti@unipr.it (M.C.A.); carlo.chezzi@unipr.it (C.C.); flora.deconto@unipr.it (F.D.C.)

**Keywords:** MALDI-TOF MS, dermatophytes identification, database

## Abstract

Despite that matrix-assisted laser desorption/ionization time-of-flight (MALDI-TOF) mass spectrometry (MS) has become a powerful tool in the clinical microbiology setting, few studies have till now focused on MALDI-TOF MS-based identification of dermatophytes. In this study, we analyze dermatophytes strains isolated from clinical samples by MALDI-TOF MS to supplement the reference database available in our laboratory. Twenty four dermatophytes (13 reference strains and 11 field isolated strains), identified by both conventional and molecular standard procedures, were analyzed by MALDI-TOF MS, and the spectra obtained were used to supplement the available database, limited to a few species. To verify the robustness of the implemented database, 64 clinical isolates other than those used for the implementation were identified by MALDI-TOF MS. The implementation allowed the identification of the species not included in the original database, reinforced the identification of the species already present and correctly identified those within the *Trichophyton mentagrophytes* complex previously classified as *Trichophyton. tonsurans* by MALDI-TOF MS*.* The dendrogram obtained by analyzing the proteic profiles of the different species of dermatophytes reflected their taxonomy, showing moreover, in some cases, a different clusterization between the spectra already present in the database and those newly added. In this study, MALDI-TOF MS proved to be a useful tool suitable for the identification of dermatophytes for diagnostic purpose.

## 1. Introduction

In the last few years, matrix-assisted laser desorption/ionization time-of-flight mass spectrometry (MALDI-TOF MS) has been increasingly studied and was originally adapted for the identification of prokaryotic organisms. New developments have extended its use to a few eukaryotic organisms, such as yeasts and molds, making this technique a straightforward, fast and reliable identification method for bacteria, yeasts and molds in a cost-effective way [1,2]. MALDI-TOF MS has been introduced as an alternative method to conventional and routine identification methods in clinical microbiological laboratories with marked success [1]. However, the assessment of MALDI-TOF for species-level identification of filamentous fungi is not as extensive, and in particular, studies examining the applications of this technique specifically for dermatophyte identification are limited (*i.e*., [3,4]).

Dermatophytes, comprising species within the genera *Epidermophyton*, *Microsporum* and *Trichophyton*, are a unique group of closely-related filamentous fungi that invade keratinized cutaneous structures, including the stratum corneum, nails and hair of humans and animals, resulting in an infection referred to as dermatophytosis, ringworm or tinea [3,5]. These superficial mycoses affect 20% to 25% of the world’s population [6].

Laboratory identification of dermatophytes is typically based on macroscopic observation of colony morphology (*i.e*., pigmentation, growth rate, texture, *etc.*) grown on selective media, followed by microscopic examination of conidia, as reported by Theel *et al.* [3]. In addition, specialized *Trichophyton* agars may be used for differentiation of species within the genera [7]. These phenotypic techniques require experienced technicians and are often labor-intensive, with a prolonged turnaround time. With the advancement of modern molecular techniques, some laboratories have turned to dermatophytes identification schemes involving sequencing of specific ribosomal DNA regions or real-time PCR amplification of discrete genes [4]. While these methods are highly accurate and rapid, they are costly and can be complex to implement. However, dermatophytes belonging to the *T. mentagrophytes* complex are particularly hard to identify on the basis of their morphological features, and the inter-species relationships within this group are unclear [4]. MALDI-TOF mass spectrometry may represent an alternative to conventional and molecular dermatophyte identification [3,4].

The aim of this study was to implement the MALDI-TOF database by using both a dermatophyte reference and clinical isolates accurately identified by conventional and molecular methods. This was done to better identify at the species level the dermatophytes producing spores.

## 2. Results and Discussion

In this study, the Bruker Daltonics database, already including 12 proteic profiles for 12 strains belonging to seven species, *Epidermophyton floccosum*, *Microsporum canis*, *Microsporum gypseum* (three), *Trichophyton rubrum* (two), *Trichophyton interdigitale*, *Trichophyton mentagrophytes* and *Trichophyton tonsurans* (three), was supplemented with 13 proteic profiles of 13 different reference strains belonging to 10 species of dermatophytes and 11 proteic profiles belonging to eight species obtained from clinical isolates identified by both conventional methods and sequencing, for a total of 13 species (Table 1). For all of the strains, except for the species *T. interdigitale* and *T. mentagrophytes*, the respective sequence analyzed confirmed the results obtained by conventional methods. In particular, as reported in Table 1, the sequences of *T*. *tonsurans* PR1, *T*. *rubrum* PR1, *M*. *canis* PR1-2, *T*. *violaceum* PR1, *T*. *soudanense* PR1 and *M*. *audouinii* PR1 fully match with *T*. *tonsurans* GenBank KF437399.1, *T*. *rubrum* GenBank KF437402.1, *M*. *canis* GenBank EU590655.1, *T*. *violaceum* GenBank KF360236.1, *T*. *soudanense* GenBank KF360237.1 and *M*. *audouinii* GenBank JN134145.1, respectively. The sequences of *T. interdigitale* PR1-2 and *T. mentagrophytes* PR1-2 match both with *T. interdigitale* GenBank KF358468.1 and *T. mentagrophytes* GenBank KF360239.1. The same result was also obtained after the analysis of the sequences of reference strains *T. interdigitale* NQ 1208, *T. interdigitale* NQ 1681 and *T*. *mentagrophytes* NQ 0866 (matching with *T. interdigitale* GenBank KF358468.1*/T. mentagrophytes* GenBank KF360239.1, *T. interdigitale* GenBank KF358466.1*/T. mentagrophytes* GenBank KF360239.1, *T. interdigitale* GenBank HG793054.1*/T. mentagrophytes* GenBank HQ223449.1, respectively).

**Figure 1:**
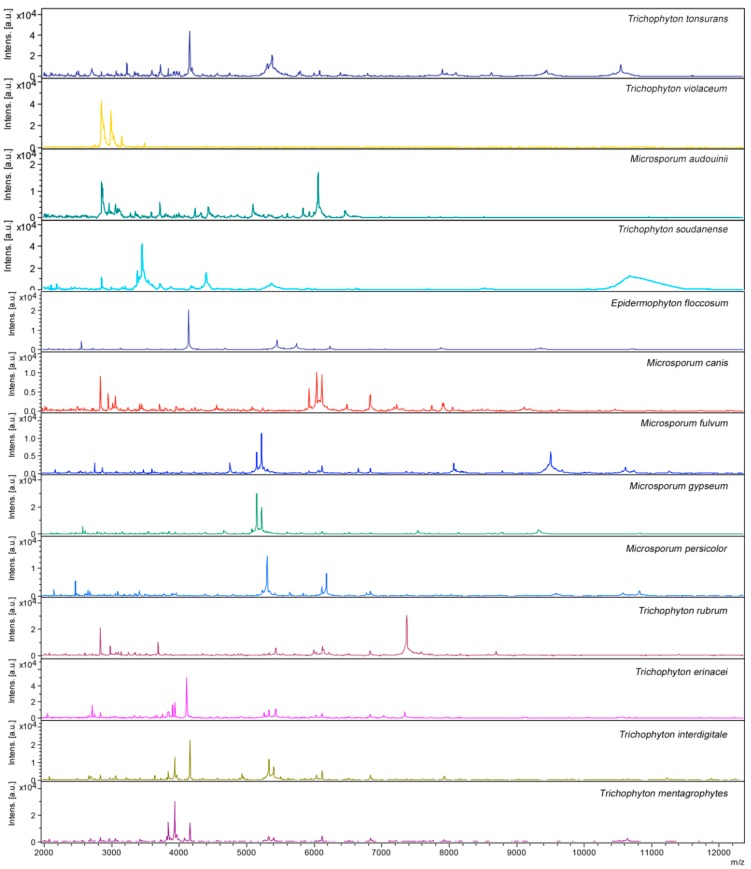
Spectra obtained by MALDI-TOF MS analysis of the 13 species of dermatophytes supplemented in the Bruker Daltonics database.

Each species used in this study to supplement the Bruker Daltonics database yielded a proteic profile, including unique peaks (Figure 1), so that each dermatophyte species analyzed gave a unique species-specific MALDI-TOF MS profile. On the contrary, the proteic profiles of *T. interdigitale* and *T. mentagrophytes* appear very similar.

Each session was validated by external negative and positive controls: in all of the experiments, the negative control spots yielded no peaks or faint profiles, which were not identifiable by the system, and the positive control spots yielded the expected *E. coli* identification with an identification score of 2–2.5. Moreover, for each strain, at least two independent experiments from two different cultures on two different days were run, and at least eight replicates/run were analyzed in order to ensure the reproducibility of the results obtained. No differences in the spectra obtained in these independent experiments were observed, demonstrating the reproducibility of the generated spectra.

When compared with the available Bruker Daltonics database, the spectra obtained from six of the 13 reference strains, *E. floccosum*, *M. canis*, *M. gypseum*, *T. tonsurans* and *T. rubrum* (two), were identified, correctly matching always with the relative proteic profiles (score value ≥ 2.0 for each of the replicates) already present in the database. The proteic profiles of *M. fulvum*, *M. persicolor* and *T. erinacei*, not included in the Bruker Daltonics database, were found to be original, matching none of the existing profiles in the database (score value < 1.3). As concerns *T. interdigitale* and *T. mentagrophytes*, the spectra obtained compared with the available Bruker Daltonics database were erroneously identified, matching always with *T. tonsurans* (score value > 2.0).

**Table 1:** Matrix-assisted laser desorption/ionization time-of-flight mass spectrometry (MALDI-TOF MS) results in comparison with conventional methods and sequencing.

Strains	ID CM ^a^	ID Sequencing	ID MALDI-TOF MS			
			Before Suppl. ^b^	Score	After Suppl. ^b^	Score
**Supplemented reference strains (13)**						
*T. tonsurans* 0865	*T. tonsurans*		*T. tonsurans*	2.01	*T. tonsurans*	2.42
*T. rubrum* 1515	*T. rubrum*	*T. rubrum*	*T. rubrum*	2	*T. rubrum*	2.35
*T. rubrum* 0544	*T. rubrum*		*T. rubrum*	2.03	*T. rubrum*	2.41
*T. interdigitale* 1681	*T. interdigitale*	*T. ment.*/*T. int.* ^c^	*T. tonsurans*	2.22	*T. interdigitale*	2.42
*T. interdigitale* 1208	*T. interdigitale*	*T. ment.*/*T. int.* ^c^	*T. tonsurans*	2.14	*T. interdigitale*	2.34
*T. interdigitale* 0389	*T. interdigitale*		*T. tonsurans*	2.05	*T. interdigitale*	2.3
*T. mentagrophytes* 0866	*T. mentagrophytes*	*T. ment.*/*T. int.* ^c^	*T. tonsurans*	2.09	*T. mentagrophytes*	2.48
*T. erinacei* 0388	*T. erinacei*		NR	<1.5	*T. erinacei*	2.27
*M. canis* 0164	*M. canis*	*M. canis*	*M. canis*	2.25	*M. canis*	2.31
*M. persicolor* 0165	*M. persicolor*		NR	<1.3	*M. persicolor*	2.29
*M. gypseum* 7177	*M. gypseum*		*M. gypseum*	2.13	*M. gypseum*	2.27
*M. fulvum* 9834	*M. fulvum*		NR	<1.6	*M. fulvum*	2.18
*E. floccosum* 1207	*E. floccosum*		*E. floccosum*	2.12	*E. floccosum*	2.35
**Supplemented clinical isolates (11)**						
*T. tonsurans* PR1	*T. tonsurans*	*T. tonsurans*	*T. tonsurans*	2.12	*T. tonsurans*	2.34
*T. rubrum* PR1	*T. rubrum*	*T. rubrum*	*T. rubrum*	2.01	*T. rubrum*	2.12
*T. interdigitale* PR1	*T. interdigitale*	*T. ment.*/*T. int.* ^c^	*T. tonsurans*	2.09	*T. ment.*/*T. int.* ^c^	2.19
*T. interdigitale* PR2	*T. interdigitale*	*T. ment.*/*T. int.* ^c^	*T. tonsurans*	2.14	*T. ment.*/*T. int.* ^c^	2.36
*T. mentagrophytes* PR1	*T. mentagrophytes*	*T. ment.*/*T. int.* ^c^	*T. tonsurans*	2.14	*T. ment.*/*T. int.* ^c^	2.19
*T. mentagrophytes* PR2	*T. mentagrophytes*	*T. ment.*/*T. int.* ^c^	*T. tonsurans*	2.18	*T. ment.*/*T. int.* ^c^	2.25
*M. canis* PR1	*M. canis*	*M. canis*	*M. canis*	1.95	*M. canis*	2.45
*M. canis* PR2	*M. canis*	*M. canis*	*M. canis*	2.05	*M. canis*	2.34
*T. violaceum* PR1	*T. violaceum*	*T. violaceum*	NR	<1.5	*T. violaceum*	2.22
*T. soudanense* PR1	*T. soudanense*	*T. soudanense*	NR	<1.3	*T. soudanense*	2.15
*M. audouinii* PR1	*M. audouinii*	*M. audouinii*	NR	<1.6	*M. audouinii*	2.21
**Identified clinical isolates (64)**						
EFPR 1, 2, 3	*E. floccosum*		*E. floccosum*	>2	*E. floccosum*	>2
MCPR 3, 4, 5, 9			*M. canis*	>2	*M. canis*	>2
MCPR 6, 7	*M. canis*		*M. canis*	1.7–2	*M. canis*	>2
MCPR 8			NR	<1.7	*M. canis*	>2
MAPR 2	*M. audouinii*		*M. canis*	1.7–2	*M. audouinii*	1.7–2
TVPR 2, 3	*T. violaceum*		NR	<1.5	*T. violaceum*	1.7–2
TTPR 3	*T. tonsurans*		*T. tonsurans*	>2	*T. tonsurans*	>2
TTPR 4			*T. tonsurans*	1.7–2	*T. tonsurans*	>2
TTPR 2			*T. rubrum*	1.7–2	*T. tonsurans*	1.7–2
TRPR 9, 16, 19, 21, 22, 23	*T. rubrum*		*T. rubrum*	>2	*T. rubrum*	>2
TRPR 2, 3, 5, 12, 14, 15, 24			*T. rubrum*	1.7–2	*T. rubrum*	1.7–2
TRPR 4, 10, 11, 17, 18, 20, 23			*T. rubrum*	1.7–2	*T. rubrum*	>2
TRPR 6, 8			*T. tonsurans*	1.7–2	*T. rubrum*	1.7–2
TRPR 7			NR	<1.7	*T. rubrum*	1.7–2
TMPR 3, 4, 5, 9, 11, 12, 14, 15, 16, 17, 18	*T. mentagrophytes*		*T. tonsurans*	>2	*T. ment.*/*T. int.* ^c^	>2
TMPR 6, 19			*T. tonsurans*	>2	*T. ment.*/*T. int.* ^c^	1.7–2
TMPR 7			*M. gypseum*	2	*T. ment.*/*T. int.* ^c^	>2
TMPR 8, 13, 20, 21			*T. tonsurans*	1.7–2	*T. ment.*/*T. int.* ^c^	>2
TMPR 10			NR	<1.5	*T. ment.*/*T. int.* ^c ^	>2
TIPR 3, 4	*T. interdigitale*		*T. tonsurans*	>2	*T. ment.*/*T. int.* ^c^	>2
TSPR 2	*T. soudanense*		*T. rubrum*	1.7–2	*T. soudanense*	1.7–2
TSPR 3			NR	<1.5	*T. soudanense*	>2
MGPR 1	*M. gypseum*		*M. gypseum*	>2	*M. gypseum*	>2
MPPR 1	*M. persicolor*		NR	<1.6	*M. persicolor*	>2

^a^ ID CM = identification by conventional methods; ^b^ Suppl. = supplementation; ^c^ *T. ment.*/*T. int.* = *T. mentagrophytes*/*T. interdigitale*; NR = no reliable identification.

The spectra of the 13 reference strains obtained by MALDI-TOF MS automatic acquisition were analyzed using Flex Analysis software (Bruker Daltonics, Bremen, Germany). The replicates with an intensity <10^4^ arbitrary units, as well as those with a profile highly different from the others were discarded. At least eight replicates for each strain were chosen and used to create a reference mean spectrum profile (MSP) through the automated function of MALDI Biotyper software (Bruker Daltonics).

The 13 MSP reference spectra obtained for the reference strains were deposited in the database for further blind identification of additional fungal strains, and their reliability was initially verified by re-analyzing all of these reference strains in a new independent experiment (score value > 2.0).

In order to increase the number of the species of dermatophytes included in the database, the MSP spectra obtained for three clinical isolates identified as *T. violaceum*, *T. soudanense* and *M. audouinii* by conventional methods and sequencing were supplemented. Moreover, taking into account that, as already reported [8], the success of species matching is dependent on the number of strain entries within the database for a given species, for eight additional clinical isolates (two *M. canis*, one *T. rubrum*, one *T. tonsurans*, two *T. mentagrophytes* and two *T. interdigitale*), identified by conventional methods and sequencing, the MSP spectra were created and added to the new in-house database.

In order to verify the robustness of the implemented database, 64 clinical isolates other than the 11 used for the implementation (three *E. floccosum*, seven *M. canis*, one *M. audouinii*, two *T. violaceum*, three *T. tonsurans*, 23 *T. rubrum*, 19 *T. mentagrophytes*, two *T. interdigitale*, two *T. soudanense*, one *M. gypseum* and one *M. persicolor*), were analyzed and identified by MALDI-TOF MS as reported in Table 1.

Furthermore, after the database implementation, the misidentification between *T. interdigitale* and *T. mentagrophytes* with *T. tonsurans* observed with the original database was avoided. In these cases, the implementation of the database was demonstrated to be particularly essential, since, despite being closely related [9,10], these species need to be distinguished, having different body areas as the target for their pathogenic action [9,10]. However, in our experience, both MALDI-TOF MS analysis and sequencing of the amplicons obtained by the PCR assay used were not able to distinguish among the species of the *T. mentagrophytes* complex, in particular between *T. interdigitale* and *T. mentagrophytes*. The identification of the species included in the *T. mentagrophytes* complex, as reported by Packeu *et al.*, is not straightforward, and morphological analysis and multilocus gene sequencing are sometimes required [4,11]. In addition, in order to differentiate the spectra of these two species, a statistical analysis by using specific software (ClinProTools version 2.0, Bruker Daltonics) was performed, and statistically, no significant difference was found.

Nevertheless, in our experience, the implementation was necessary in order to have the possibility of identification of the dermatophytes species more frequently isolated in our area.

The strength of the performed implementation was also supported by the simplicity of the protein extraction method.

**Figure 2:**
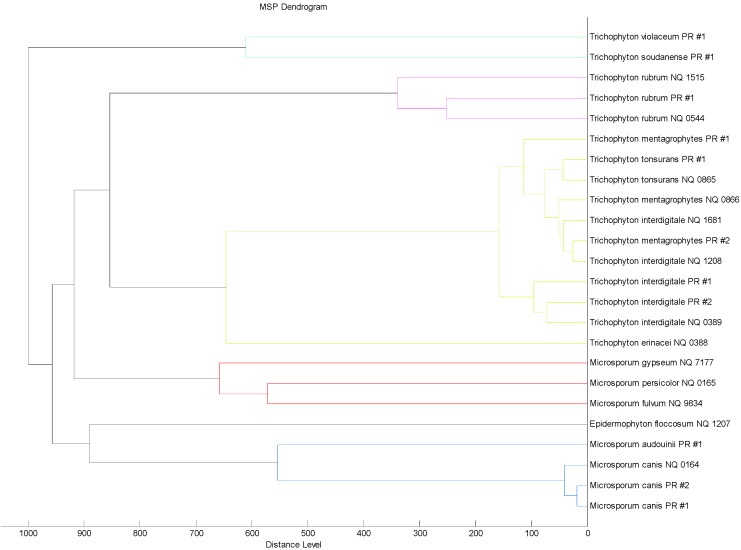
“Main spectra profiles”-based (MSP) dendrogram of the 24 dermatophytes strains supplemented in the new in-house database.

In order to evaluate the similarity among the MSP reference spectra of the 24 dermatophytes strains supplemented in the new in-house database, an MSP spectra-based dendrogram was created (Figure 2). The dendrogram revealed a clusterization that reflects the phylogenetic taxonomic tree of the dermatophytes reported by De Hoogh *et al.* [10]. However, *T. interdigitale* and *T.* *mentagrophytes* MSP spectra present in the dendrogram are closely related and do not give rise to two separate clusters, showing high similarity among the spectra belonging to these two species. For this reason, MALDI-TOF MS failed to distinguish between *T. interdigitale* and *T.* *mentagrophytes*, as well as the identification based on their ITS sequence performed in this study.

**Figure 3:**
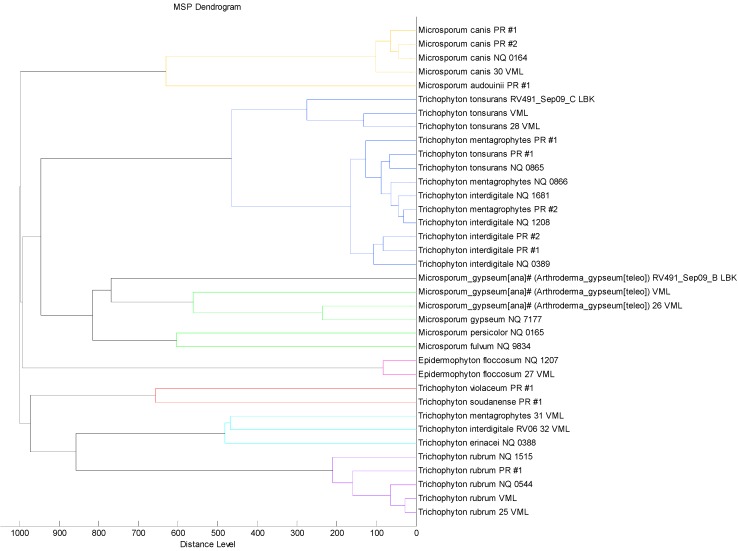
“Main spectra profiles”-based dendrogram of the 12 proteic profiles already included in Bruker Daltonics Database and 24 dermatophytes strains supplemented in the new in-house database.

Moreover, the dendrogram created with the 12 proteic profiles already included in the Bruker Daltonics database and the 24 dermatophytes strains supplemented in the new in-house database (Figure 3) does not reproduce the phylogenetic taxonomic tree for the genus *Trichophyton* [10]. In this case, as expected, the MSP spectra of the strains belonging to the species, *E. floccosum*, *T. rubrum*, *T. tonsurans*, *M. canis* and *M. gypseum*, already present and supplemented in the database, are very similar and then cluster closely; on the contrary, the MSP spectra of the strains belonging to the species, *T. interdigitale* and *T. mentagrophytes*, present in the Bruker Daltonics database and those added in this study, cluster separately. This result could explain the different identification performed by the system before and after the implementation of the database (Table 1). This result was not affected by the fungal growth condition used in this study, since, in our experience, no differences were observed in the spectra obtained by the analysis of proteins extracted from molds grown in solid cultures or in liquid cultures, as suggested by Bruker Daltonics (data not shown). Moreover, the extraction protocol described in this study allows a rapid fungal identification, since it is performed directly from solid culture, avoiding a sub-culture step in liquid medium.

## 3. Experimental Section

### 3.1. Fungal Strains and Culture Conditions

A total of 13 reference U.K. NEQAS (The United Kingdom National External Quality Assessment Service for Microbiology) strains, namely *Epidermophyton floccosum*, *Microsporum canis*, *M. gypseum*, *M. fulvum*, *M. persicolor*, *Trichophyton rubrum* (2), *T. interdigitale* (3), *T. erinacei*, *T. mentagrophytes* and *T*. *tonsurans*, identified by conventional methods and some of them also by sequencing, were included in this study. Furthermore, 11 clinical isolates (2 *M. canis*, 1 *T. rubrum*, 1 *T. tonsurans*, 1 *T. violaceum*, 1 *T. soudanense*, 1 *M. audouinii*, 2 *T. mentagrophytes* and 2 *T. interdigitale*) identified by conventional standard procedures [10] and sequencing, were also included.

In addition, to validate the new in-house database, 64 clinical isolates (3 *E. floccosum*, 7 *M. canis*, 1 *M. audouinii*, 2 *T. violaceum*, 3 *T. tonsurans*, 23 *T. rubrum*, 19 *T. mentagrophytes*, 2 *T. interdigitale*, 2 *T. soudanense*, 1 *M. gypseum* and 1 *M. persicolor*), different from those used for the implementation and identified by conventional standard procedures [10], were analyzed and identified by MALDI-TOF MS (Bruker Daltonics, Bremen, Germany).

The fungal strains used in this study were cultured on Sabouraud’s dextrose agar plates with chloramphenicol (Kima, Padua, Italy), at 30 °C for 3 weeks, in order to obtain typical morphological characteristics [10].

### 3.2. DNA Extraction

DNA from cultivated fungi (5 reference strains, *T. rubrum* 1515, *T. interdigitale* 1681, *T. interdigitale* 1208, *T. mentagrophytes* 0866, *M. canis* 0164 and all of the 11 clinical isolates implemented in the MALDI-TOF database) was extracted using the High Pure PCR Template Preparation kit (Roche Diagnostics, Mannheim, Germany), according to the manufacturer’s instructions. Extracted DNA was used immediately for PCR assays, as previously described [12,13].

### 3.3. Conventional PCR and Sequencing

Purified DNA from all of the 16 strains was used for the amplification of the ribosomal DNA 5.8S/ITS2 regions, using the universal fungal primer set ITS3 and ITS4 in the same conditions previously described [13,14].

For all of the amplicons, sequencing was performed by Roche Diagnostics (TIB Molbiol S.r.l., Genova, Italy). The sequences were aligned using Molecular Evolutionary Genetics Analysis (MEGA) software version 6.06 [15] and compared with deposited sequences of dermatophytes in GenBank.

### 3.4. Sample Preparation for Matrix-Assisted Laser Desorption/Ionization Time-of-Flight Mass Spectrometry (MALDI-TOF MS) Analysis

Fungal analysis by MALDI-TOF MS requires chemical extraction of the isolated organism. Briefly, a swab moistened with sterile H_2_O was smeared on the surface of the mycelium and re-submerged to obtain a 3-mL turbid solution. After a centrifugation for 2 min at 17,500× *g*, the obtained pellet was washed in double-distilled water and centrifuged in the same conditions; the supernatant was removed, and the pellet was re-suspended in 300 μL of double-distilled water and 900 μL of absolute ethanol. After centrifugation in the same conditions, the supernatant was discarded, and the obtained pellet was air-dried at least 10 min to evaporate the ethanol residue. Subsequently, the pellet was re-suspended with 50 μL of 70% formic acid and after 30 min with 50 μL of acetonitrile. This suspension was vortexed for 1 min and centrifuged again at 17,500× *g* for 2 min.

In each session, one drop (1 μL) of the supernatant obtained by the formic acid/acetonitrile protein extraction was transferred in a unique deposition on the polished steel MSP-96 target plate (Bruker Daltonics), accurately washed, as previously described [8]. After drying at room temperature for about 10 min, 1 μL of a saturated α-cyano-4-hydroxy-cinnamic acid (HCCA) (2.5 mg) matrix solution (Bruker Daltonics) re-suspended as previously described [8] was added on the plate in a unique deposition.

### 3.5. MALDI-TOF MS Analysis

The acquisition and the analysis of mass spectra were performed by a Microflex LT mass spectrometer (Bruker Daltonics) using MALDI Biotyper Software package (version 3.1) with the reference database (version 3.1.2.0; 3995 database entries, Bruker Daltonics) and default parameter settings (positive linear mode; laser frequency 60 Hz; ion source 1 voltage, 20 kV; ion source 2 voltage, 16.7 kV; lens voltage, 7.0 kV; mass range, 2000 to 20,000 Da).

For each spectrum 240 laser shots in 40-shot steps from different positions of the sample spot were accumulated and analyzed (automatic mode, default setting), according to the manufacturer’s procedure (Bruker Daltonics).

In each experiment, the Bruker Bacterial Test standard (BTS) (Bruker Daltonics) for calibration was used according to the instructions of the manufacturer. Moreover, in each session, *Escherichia coli* (ATCC 8739) (as a positive control) and a non-inoculated-matrix solution (as a negative control), subjected to the same protein extraction procedure, were analyzed.

As we have previously described [8], MALDI Biotyper software (Bruker Daltonics) compares each sample mass spectrum to the reference mass spectra present in the database using a pattern matching approach, which is based on statistical multi-variant analysis and includes peak positions and intensities; it calculates an arbitrary unit score value comprised between 0 and 3, reflecting the similarity between the sample and reference spectrum, and, finally, displays the top ten matching database records. As specified by manufacturer, an identification score ≥2.0 was accepted for a reliable identification at the species level and a score ≥1.7 and ≤2.0 for an identification at the genus level. Scores < 1.7 were considered unreliable.

### 3.6. MALDI-TOF MS Database for Dermatophytes

The reference database (Bruker Daltonics; version 3.1.2.0) available in our laboratory already comprised a set of 12 strains belonging to 7 clinically relevant species of dermatophytes (1 *E. floccosum*, 1 *M. canis*, 3 *M. gypseum*, 2 *T. rubrum*, 1 *T. interdigitale*, 1 *T. mentagrophytes*, 3 *T. tonsurans*).

For each of the 24 strains analyzed in this study and supplemented in the Bruker Daltonics database, 16 replicates were analyzed by MALDI-TOF MS. The respective obtained spectra were analyzed by the FlexAnalysis software (Version 3.0, Bruker Daltonics) to carry out “Smoothing” ,“Baseline” and to select spectra with an intensity <10^4^ arbitrary units and single spectra with peaks differing from the other. These selected spectra were removed, and the remaining were used to calculate a reference mean spectrum profile (MSP), by the automated function of MALDI Biotyper software (Biotyper MSP Creation Standard Method, Bruker Daltonics), as previously described [16], created completely unsupervised by extracting information about peak position, intensity and frequency based on an unbiased algorithm. The MSP spectra were used for MALDI Biotyper database implementation.

### 3.7. MALDI-TOF MS Dendrogram

Cluster analysis was performed based on the comparison of each strain-specific main spectra (MSP dendrogram) created, as we have previously described in two papers [16,17]. The parameter settings were: “Distance Measure Euclidian” and “Linkage complete”. The linkage function is normalized according to the distance between 0 (perfect match) and 1000 (no match).

## 4. Conclusions

Despite MALDI-TOF mass spectrometry having become a powerful tool in the clinical microbiology setting, enabling the rapid identification of bacteria and yeasts for laboratory diagnosis, few studies have till now focused on MALDI-TOF-based identification of dermatophytes (*i.e*., [3,4,5,18,19]).

In our laboratory, too, the lack in the Bruker Daltonics database as concerns dermatophytes led us to supplement it with further proteic profiles from some strains of these fungi, obtained by using an easy-to-perform protocol of protein extraction. This work followed the suggestion of some authors [20] to expand the MALDI-TOF database with some fungal group of clinical importance in order to help to enhance the utility of this methodology for the identification of unknown fungal pathogens.

As already demonstrated in our laboratory for bacterial identification by MALDI-TOF MS [8,16], in this study, this technique proved to be a useful tool that could be suitable for application in the identification of fungi, both for diagnostic purposes and epidemiological surveillance, and we hope that the new spectra could be available freely to the scientific community through a web platform.
